# Apoptotic Fragmentation of Tricellulin

**DOI:** 10.3390/ijms20194882

**Published:** 2019-10-01

**Authors:** Susanne Janke, Sonnhild Mittag, Juliane Reiche, Otmar Huber

**Affiliations:** Department of Biochemistry II, Jena University Hospital, Friedrich Schiller University Jena, 07743 Jena, Germany; susanne.janke@med.uni-jena.de (S.J.); sonnhild.mittag@med.uni-jena.de (S.M.); juliane.reiche@med.uni-jena.de (J.R.)

**Keywords:** tight junction, tricellulin, lipolysis-stimulated lipoprotein receptor (LSR), angulin, epithelial barrier, cell–cell contact, apoptosis, caspase

## Abstract

Apoptotic extrusion of cells from epithelial cell layers is of central importance for epithelial homeostasis. As a prerequisite cell–cell contacts between apoptotic cells and their neighbors have to be dissociated. Tricellular tight junctions (tTJs) represent specialized structures that seal polarized epithelial cells at sites where three cells meet and are characterized by the specific expression of tricellulin and angulins. Here, we specifically addressed the fate of tricellulin in apoptotic cells. Methods: Apoptosis was induced by staurosporine or camptothecin in MDCKII and RT-112 cells. The fate of tricellulin was analyzed by Western blotting and immunofluorescence microscopy. Caspase activity was inhibited by Z-VAD-FMK or Z-DEVD-FMK. Results: Induction of apoptosis induces the degradation of tricellulin with time. Aspartate residues 487 and 441 were identified as caspase cleavage-sites in the C-terminal coiled-coil domain of human tricellulin. Fragmentation of tricellulin was inhibited in the presence of caspase inhibitors or when Asp487 or Asp441 were mutated to asparagine. Deletion of the tricellulin C-terminal amino acids prevented binding to lipolysis-stimulated lipoprotein receptor (LSR)/angulin-1 and thus should impair specific localization of tricellulin to tTJs. Conclusions: Tricellulin is a substrate of caspases and its cleavage in consequence contributes to the dissolution of tTJs during apoptosis.

## 1. Introduction

Tight junctions (TJs) represent the most apical cell–cell contacts in polarized epithelial cell layers. They form essential barriers for solutes including nutrients, metabolites and toxins as well as ions, thereby separating luminal and external compartments from the interior of multicellular bodies. Accordingly, TJs are also important to exclude pathogens such as bacteria and viruses from organisms. Break-down of this barrier function is associated with different diseases and is involved in inflammatory bowel diseases [1] or in organ failure during sepsis [2,3]. However, it turned out that TJ components may also represent targets of specific bacteria and viruses to invade cells or tissues resulting in a deregulation of the cytoskeleton or of signaling pathways required for barrier maintenance [4]. Primarily, claudins and occludin form the apical sealing belt between two opposing cells where they are arranged in a network of TJ strands that restricts and regulates paracellular flux depending on the specific claudin composition. The situation is different at tricellular contacts where apical TJs strands of three neighboring cells meet and thus are assumed to represent weak points for the paracellular TJ network [5]. At these tricellular tight junctions, bicellular TJs converge, and TJ strands turn basally and form the so-called central sealing element [6]. These tricellular TJs (tTJs) differ from bicellular TJs (bTJs) by a specific set of proteins including tricellulin [7] and one of the three angulins [8].

Tricellulin (marvelD2) together with occludin and marvelD3 form the TJ-associated MARVEL protein (TAMP) family of 4-transmembrane-domain proteins with long N- and C-terminal domains both located in the cytosol. The tricellulin C-terminus interacts with the cytosolic adapter protein zona occludens-1 (ZO-1) and is thereby linked to the actin cytoskeleton [9,10,11]. Knock-down of tricellulin impairs epithelial barrier function and results in disorganized bTJs, indicating an essential role of tricellulin in maintenance of overall TJ structure and function [7]. Tricellulin at tTJs was shown to restrict macromolecular passage and strong overexpression contributes to reduced paracellular permeability [12,13]. Interestingly, in occludin knock-down cells, tricellulin is mislocated to bTJs preferentially at edges of elongating bTJs [14]. How homomeric or heteromeric tricellulin/occludin complexes may be involved in this process or in the transport of tricellulin to the TJs [15] is currently not understood. However, it is obvious that tricellulin and occludin do not form heterophilic trans-interactions within established TJs [11,16].

With the identification of LSR (lipolysis-stimulated lipoprotein receptor)/angulin-1 as a tTJ-located receptor that recruits tricellulin to tTJs, it became clear that a specific anchor–protein is responsible for the defined localization of tricellulin. Knock-down of LSR/angulin-1 impaired barrier function in reducing transepithelial resistance (TER) probably as a consequence of mislocalization of tricellulin to bTJs [17]. In addition, mislocalization of LSR was associated with cancer progression and metastasis [18]. Based on sequence homology, two LSR/angulin-1-related proteins including immunoglobulin-like domain-containing receptor (ILDR) 1/angulin-2 and ILDR 2/angulin-3 were identified. The three members show tissue-specific expression patterns and at least one family member is located at tTJs [8,19]. However, it is still open regarding how angulins are directed to tTJs.

The physiological importance of tricellulin for hair cells is emphasized by mutations in the tricellulin C-terminal domain found in patients with nonsyndromic deafness (DFNB49). All mutations result in tricellulin variants with premature stop codons and, consequently, structurally impaired C-terminal ends and limited binding to ZO-1 [20,21]. Moreover, truncation of the tricellulin C-terminal domain diminishes its correct localization to tricellular contacts [22]. Tricellulin knock-out mice show a progressive hearing loss due to apoptotic death of hair cells [23]. It is currently not clear why tricellulin knock-out mice show this limited and hair cell-specific phenotype although tricellulin was deleted in all other tissues too. Interestingly, ILDR1/angulin-2 deficiency causes a similar phenotype with progressing hearing loss by outer hair cell degeneration [19,24,25]. Whether compensatory effects or regulatory roles of tricellulin and/or angulins may play a role still has to be solved.

Little is known in respect to mechanisms involved in regulation of tricellulin function. Like occludin, tricellulin can be phosphorylated [7]. However, in occludin, a multitude of kinases and corresponding phosphosites as well as functional consequences of these modifications have been studied in detail [26]. A phosphorylation hotspot region [27] that seems to be involved in the regulation of occludin mobility, apical junctional complex dynamics and cell migration [28] has been identified. In contrast, tricellulin phosphorylation-sites have been found by mass spectrometry, but, to our knowledge, none of them has been functionally characterized. However, there is the first evidence that occludin and tricellulin are differentially targeted by specific kinases [29]. Interestingly, JNK1/JNK2-dependent phosphorylation of LSR/angulin-1 was reported to be crucial for its exclusive localization to tTJs [30]. In addition, tricellulin was shown to be targeted by the ubiquitin ligase Itch, which might affect its stability and trafficking [31].

To maintain epithelial functionality, old or damaged cells have to be removed by apoptosis and replaced by new cells. Apoptosis is induced by extrinsic and intrinsic signals. Both pathways in an initial step lead to activation of initiator caspases, which subsequently activate effector caspases. These finally hydrolyze essential structural and housekeeping proteins by cleavage after aspartate residues [32]. Caspases have multiple functions also in inflammation and immunity [33]. Extrusion of apoptotic cells from the cell layer ideally occurs without loss of barrier function and disruption of the cytoarchitecture [34]. In consequence, contacts between the dying and neighboring cells have to be disassembled. Previous studies have analyzed bTJs in this respect [35], but, to our knowledge, tTJs have not been investigated in more detail. Here, we induced apoptosis with staurosporine or camptothecin and observed a caspase-dependent cleavage of the C-terminus of tricellulin. We identified two specific caspase cleavage-sites and mutation of these sites protected tricellulin from fragmentation. In addition, we provide evidence that LSR/angulin-1, which anchors tricellulin to tTJs, is also targeted by caspases.

## 2. Results

### 2.1. Caspase-Dependent Cleavage of Tricellulin upon Apoptotic Stimuli

**Figure 1:**
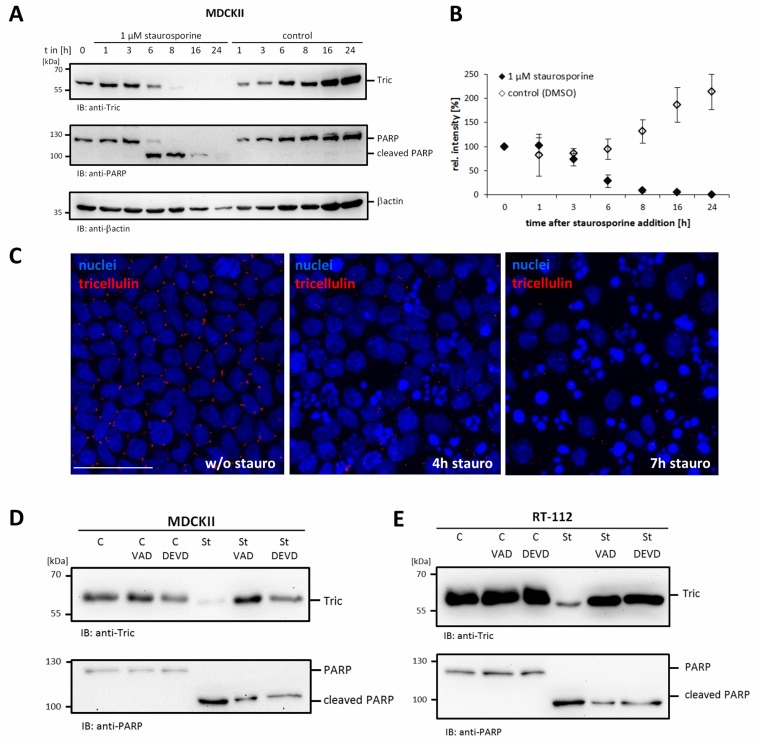
Tricellulin is a target of caspase cleavage in apoptotic cells. (**A**) MDCKII cells were treated with 1 µM staurosporine or DMSO as solvent control for the indicated times. Cell lysates were analyzed by Western blotting using anti-tricellulin, anti-poly[ADP-ribose] polymerase (PARP) and anti-βactin antibodies. (**B**) Quantification of tricellulin degradation by densitometric analysis. The graph represents mean values +/− SD of three independent experiments. (**C**) Loss of endogenous tricellulin detected by immunofluorescence microscopy (scale bar 30 µm). (**D**) Pre-treatment with 10 µM Z-VAD-FMK or 20 µM Z-DEVD-FMK for 1 h before stimulation with 1 µM staurosporine inhibited tricellulin degradation. Lysates were generated 6 h after induction of apoptosis. (**E**) Staurosporine-induced cleavage of tricellulin in RT-112 cells is inhibited by caspase inhibitors (10 µM Z-VAD-FMK or 20 µM Z-DEVD-FMK). All experiments are representatives of at least three independent experiments.

To investigate the fate of tricellulin during apoptosis, MDCKII cells were treated with staurosporine as a known inducer of apoptosis [36]. At different time points after addition of staurosporine, both floating and adherent cells were collected and lysed for subsequent Western blot analysis. Already three hours after induction of apoptosis, a time-dependent decline of endogenous tricellulin was detectable compared to DMSO-treated control cells (Figure 1A,B). Induction of apoptosis was confirmed by detection of cleaved poly(ADP-ribose)-polymerase (PARP), a well-known effector caspase substrate. The typical PARP cleavage fragment was detectable only in staurosporine-treated cells but not in control cells (Figure 1A). Moreover, when camptothecin was used as an alternative apoptosis inducer, the loss of endogenous tricellulin in MDCKII cells was detectable between 8 h and 24 h (Supplementary Figure S1). The produced tricellulin cleavage fragments were not detectable using the anti-tricellulin monoclonal antibody (clone 54H19L38). Immunofluorescence microscopy confirmed degradation of tricellulin. Signals for endogenous tricellulin disappeared with time (Figure 1C).

From our observations, we hypothesized that tricellulin like occludin [35] is a caspase target during apoptosis. To verify that the loss of tricellulin is indeed the consequence of caspase cleavage MDCKII cells were pre-treated with the pan-caspase inhibitor Z-VAD-FMK or the caspase-3 inhibitor Z-DEVD-FMK before adding staurosporine. Both caspase inhibitors significantly reduced degradation of endogenous tricellulin (Figure 1D). Inhibition of caspase activity was confirmed by the reduced formation of the PARP cleavage-fragment. A similar effect was obtained in RT-112 cells (bladder carcinoma) (Figure 1E) showing that the observed effect is not restricted to MDCKII cells.

### 2.2. Mapping of Potential Caspase Cleavage-Sites in Tricellulin

**Figure 2:**
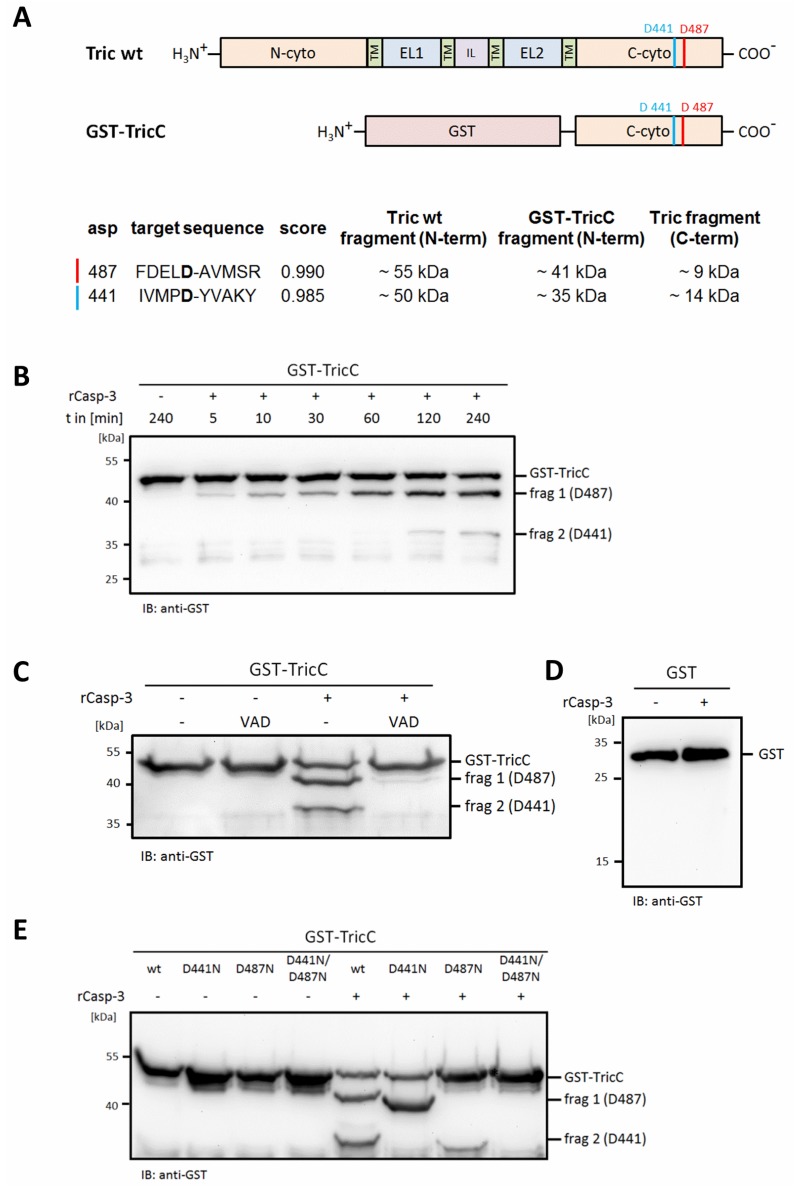
Identification of D487 and D441 as potential caspase-3 cleavage-sites in human tricellulin. (**A**) Schematic overview of the full-length tricellulin structure including the four transmembrane domains (TM), extracellular loops 1 and 2 (EL1, EL2), the intracellular loop (IL) and the potential caspase cleavage-sites D487 and D441. The table summarizes the molecular masses of the corresponding caspase cleavage-products expected for an in vitro caspase assay using recombinant GST-TricC fusion protein as substrate. (**B**) Time-dependent generation of the GST-TricC cleavage-products frag 1 and frag 2 after addition of recombinant caspase-3. The image is a representative of *n* = 2. (**C**) In the presence of pan-caspase inhibitor Z-VAD-FMK, fragmentation of GST-TricC is inhibited. (**D**) GST was used as a control and was not fragmented by caspase-3. (**E**) Mutation of potential caspase-3 cleavage-sites disable cleavage of GST-TricC partly (GST-TricC-D441N, GST-TricC-D487N) or completely (GST-TricC-D441N/D487N).

For prediction of possible caspase cleavage-sites in human tricellulin, the online software tool CaspDB [37] was applied. Two potential caspase cleavage-sites with the highest scores were predicted in the C-terminal domain of human tricellulin after amino acid aspartate 487 (D487) and/or aspartate 441 (D441) (Figure 2A). To confirm caspase-3-mediated cleavage in the C-terminal domain of tricellulin, we expressed and purified a GST-TricC fusion protein as reported previously [29,31] and applied in vitro digestion using recombinant active caspase-3. In the presence of caspase-3, two additional fragments with a molecular mass of about 40 and 35 kDa were detectable by Western blot analysis, representing potential caspase-3 cleavage-products. The molecular mass of these fragments coincides with the predicted molecular weight of GST-TricC fragments when cleaved at D487 (frag1, ~41 kDa) and D441 (frag2, ~35 kDa) (Figure 2B). Remarkably, fragment 1 is generated earlier and more efficiently than fragment 2, suggesting that D487 is the preferred caspase-3 cleavage-site and D441 appears to be used as a sequential second site after cleavage at D487. Addition of the pan-caspase inhibitor Z-VAD-FMK inhibited the generation of both GST-TricC fragments (Figure 2C). As control, purified recombinant GST alone was analyzed for caspase-3 cleavage, but no fragmentation was detectable (Figure 2D). Finally, we generated GST-TricC-D487N, GST-TricC-D441N and GST-TricC-D441N/D487N double mutant constructs and subjected them to in vitro cleavage-assays with recombinant caspase-3. Consistent with our previous results, caspase cleavage of GST-TricC-D487N only resulted in formation of fragment 2 (frag2), whereas cleavage of GST-TricC-D441N only generated fragment 1 (frag1). The GST-TricC-D441N/D487N double-mutated construct was no longer cleaved by recombinant caspase-3 (Figure 2E).

**Figure 3:**
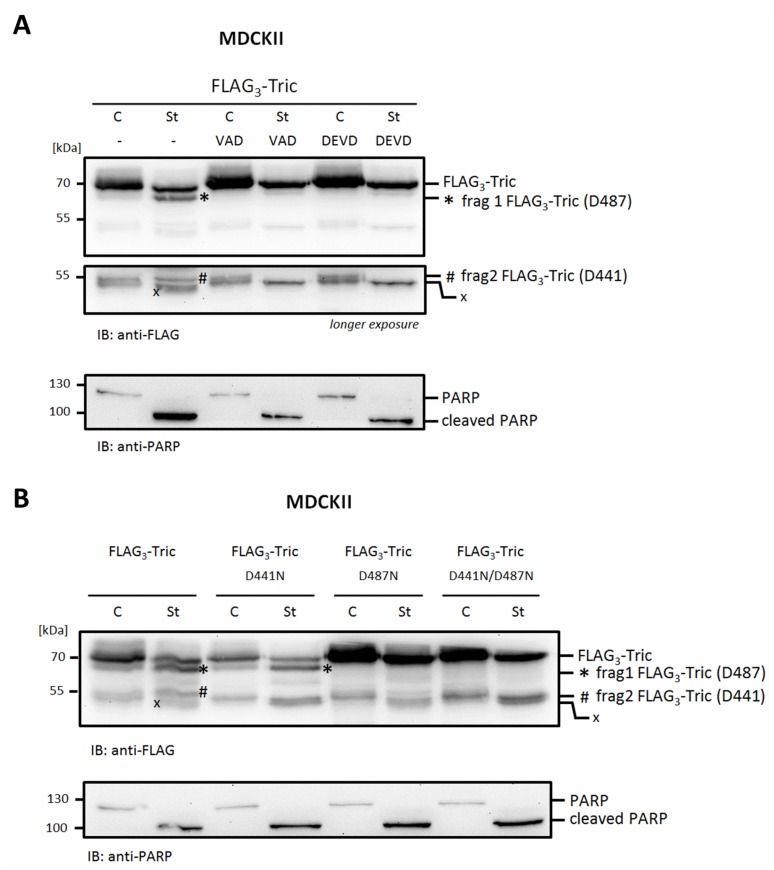
Caspase-3-mediated cleavage of FLAG_3_-tricellulin in MDCKII upon apoptosis induction. (**A**) MDCKII cells were transiently transfected with p3xFLAG-CMV10-tricellulin, pre-treated with caspase inhibitors Z-VAD-FMK (VAD) or Z-DEVD-FMK (DEVD) for 1 h before induction of apoptosis with 1 µM staurosporine for 6 h. (**B**) MDCKII cells transiently transfected with FLAG_3_-tricellulin wild-type or caspase-site mutated constructs as indicated were treated with 1 µM staurosporine for 6 h. The lower panels in (**A**) and (**B**) show Western blot detection of the typical PARP fragment generated by caspases confirming induction of apoptosis. Representative images of at least three independent experiments are shown. Bands marked with * represent caspase-dependent cleavage product frag 1 (~65 kDa) and # represents frag 2 (~55 kDa). The other bands (x) represent undefined or at least caspase-independent fragments.

To validate the in vitro results, N-terminally FLAG_3_-tagged human tricellulin was transiently transfected into MDCKII cells. Cells were subsequently treated with or without staurosporine for 6 h in the presence or absence of pan-caspase inhibitor Z-VAD-FMK and the caspase-3 inhibitor Z-DEVD-FMK, respectively. After induction of apoptosis, two bands with a molecular weight of about 55 kDa and 65 kDa were detectable (Figure 3A). Fragmentation was abrogated in the presence of each of the caspase inhibitors. In contrast to wildtype FLAG_3_-Tric, transient transfection of a mutated FLAG_3_-Tric-D441N construct abolished the generation of caspase-3 cleavage product frag 2 (~ 55 kDa) upon induction of apoptosis with staurosporine. Generation of cleavage-product frag 1 (~ 65 kDa) was not affected. Transfection of mutated FLAG_3_-Tric-D487N revealed no fragment 1 and only to a very limited amount fragment 2. When the double-mutated FLAG_3_-Tric-D441N/D487N was transfected, none of the fragments was detectable. These observations suggest that cleavage at D487 supports caspase-3-mediated cleavage at D441 (Figure 3B). Taken together, these results confirm D487 and D441 as potential caspase-sites in human tricellulin that are targeted in apoptotic cells.

### 2.3. The Functional Interaction of Tricellulin and LSR Is Disrupted during Apoptosis

**Figure 4:**
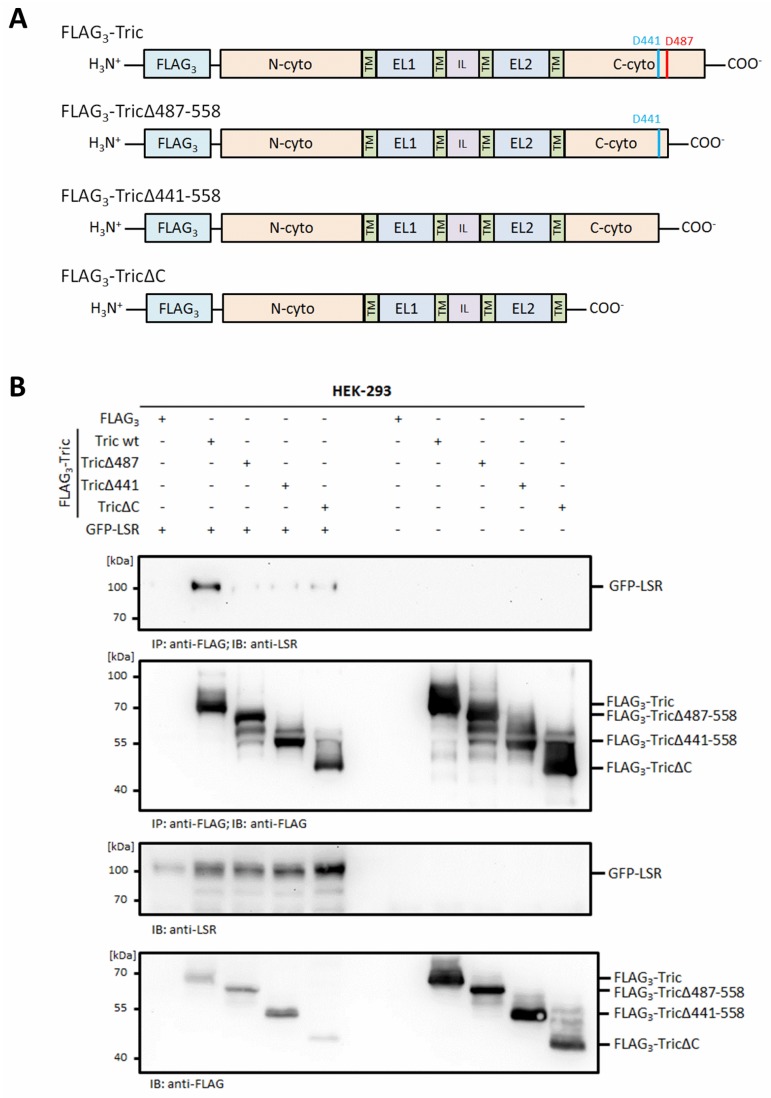
Deletion of the tricellulin C-terminus impairs binding to LSR/angulin-1. (**A**) Schematic representation of the FLAG_3_-tricellulin constructs used in the transient transfection experiments. TM, transmembrane domain; EL, extracellular loop; IL, intracellular loop. (**B**) HEK-293 cells were transiently transfected with FLAG_3_-tricellulin constructs missing the C-terminal tails corresponding to a cleavage by caspases together with GFP-LSR as indicated. Cells were lysed 48 h after transfection. Immunoprecipitaion (IP) was performed with monoclonal anti-FLAG antibody. Protein complexes from IP (upper two blots) and cell lysates (lower two blots) were analyzed by Western blotting with anti-LSR and anti-FLAG antibodies. The images are representatives of at least three independent experiments.

Tricellulin is recruited to tTJs by lipolysis-stimulated lipoprotein receptor (LSR/angulin-1) in epithelial and endothelial cells [38,39]. This interaction is mediated by the cytosolic C-terminus of tricellulin [17]. In this context, the question arises if caspase-mediated cleavage within the cytosolic C-terminus of tricellulin affects its interaction with LSR. Therefore, co-immunoprecipitation experiments were performed using cell lysates obtained from HEK-293 cells transiently transfected with LSR together with either full-length tricellulin or deletion constructs lacking amino acids 487–558 (FLAG_3_-Tric∆487–558), amino acid 441–558 (FLAG_3_-Tric∆441–558) or complete cytosolic C-terminus (FLAG_3_-Tric∆C) (Figure 4A). Confirming literature, co-transfection of FLAG_3_-Tric and green fluorescent protein GFP-tagged LSR in HEK-293 cells revealed an interaction of both proteins in co-immunoprecipitation experiments, whereas FLAG_3_-Tric∆C did only show a weak signal for GFP-LSR (Figure 4B). Similar to FLAG_3_-Tric∆C, an interaction between GFP-LSR and FLAG_3_-Tric∆487–558 or FLAG_3_-Tric∆441–558 protein lacking the cytosolic C-terminal parts released by caspases was not detectable (Figure 4B). In this context, it is interesting to note that, in a Western blot experiment, using the anti-tricellulin (clone 54H19L38) ABfinity^TM^ rabbit monoclonal antibody generated against amino acids 369–558 of human tricellulin did no longer detect neither the FLAG_3_-Tric∆487–558 nor the FLAG_3_-Tric∆441–558 protein in transiently transfected cells, thus suggesting that the epitope of this antibody is located between amino acids 487–558 (Supplementary Figure S2).

**Figure 5:**
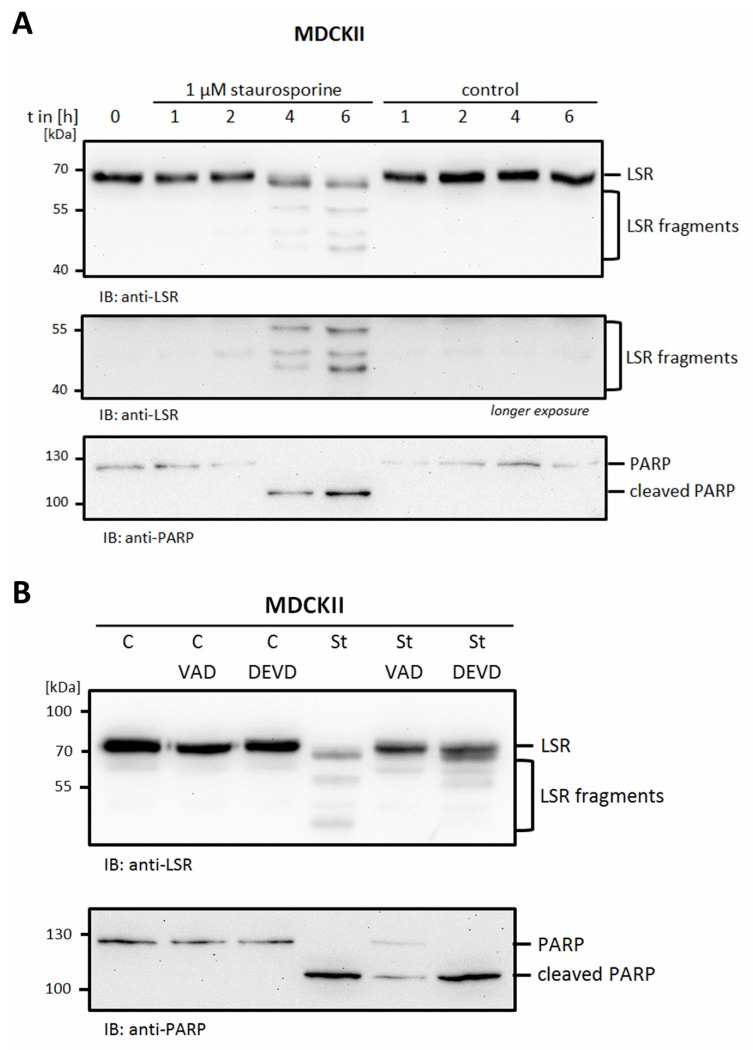
LSR in apoptotic cells. (**A**) Treatment of MDCKII cells with 1 µM staurosporine or solvent control for different times as indicated induced fragmentation of LSR. (**B**) Pre-treatment with caspase inhibitors (10 µM Z-VAD-FMK or 20 µM Z-DEVD-FMK for 1 h) inhibited fragmentation induced by staurosporine (1 µM for 6 h). Cell lysates were analyzed by Western blotting with anti-LSR and anti-PARP antibodies. Representative images of three independent experiments are shown.

These results indicate that caspase-mediated cleavage of tricellulin liberates it from angulin and thus from tTJs. However, this does not exclude that LSR/angulin-1 itself is targeted during apoptosis. Therefore, we next analyzed the fate of LSR/angulin-1 after induction of apoptosis in MDCKII cells. Indeed, staurosporine treatment led to a fragmentation of endogenous LSR/angulin-1 with time. Already 4 h after induction of apoptosis, four fragments of LSR were detectable using an anti-LSR antibody targeting the cytosolic part of LSR (Figure 5A). LSR/angulin-1 cleavage during apoptosis was inhibited by both caspase inhibitors Z-VAD-FMK and Z-DEVD-FMK. This indicates that LSR fragmentation is a consequence of caspase activation during apoptosis (Figure 5B).

## 3. Discussion

Release of apoptotic cells from epithelial and endothelial layers is critical for the maintenance of the barrier function and is often accompanied with a corresponding local decrease of transepithelial resistance [40]. In the gut, it is of importance that apoptotic cells, which are continuously extruded from the epithelium, do not generate microbial entry sites. Thus, it was not surprising that, in epithelial cell layers, there are mechanisms that efficiently break-down cell–cell contacts between apoptotic and neighboring cells. In previous studies, it was shown that components of the cadherin-catenin adhesion complex are targets of caspases. The adherens junction proteins E- and VE-cadherin are both cleaved by metalloproteinases, resulting in a release of the extracellular domain and thereby disrupting their trans-interactions with cadherin molecules on the surface of neighboring cells. Caspases are responsible for the cleavage of the cytosolic domains, thus disconnecting the interaction with the actin cytoskeleton [36,41]. Moreover, β-catenin as a linker between E-cadherin and the actin cytoskeleton is a caspase target itself [42]. Along with the adherens junctions, desmosomal cadherins and components of the cytosolic desmosomal plaque are inactivated similarly during apoptosis [43]. In addition, occludin, ZO-1 and ZO-2 were identified as caspase substrates [35]. Here, we extended this study to tricellulin and show that the C-terminal cytosolic tail of tricellulin is also targeted by caspases independent from whether apoptosis is induced by staurosporine or camptothecin and also independent from the cell system, in that case MDCKII or RT-112 cells. We identified two cleavage sites in tricellulin and mapped them C-terminal to Asp441 and Asp487. Specificity of cleavage was verified by treatment with caspase inhibitors and by mutation of the corresponding aspartate to asparagine, preventing the formation of cleavage fragments both in apoptotic cells as well as in in vitro fragmentation assays.

From our experiments, we conclude that D487 is the preferred cleavage-site. This is based on the observation that the corresponding cleavage-fragment was detectable far earlier as compared to the product generated by cleavage at D441 in in vitro cleavage-assays (Figure 2B). Moreover, it seems that initial cleavage at D487 is prerequisite for efficient cleavage at D441. This was confirmed by analyzing FLAG-tagged tricellulin where caspase cleavage-sites were mutated. The induction of apoptosis in cells transfected with wildtype FLAG_3_-tricellulin revealed two cleavage fragments. However, in cells transfected with FLAG_3_-tricellulin-D487N, a fragment generated by cleavage at D441 was more or less not detectable, most likely due to the lack of the initial cleavage at D487 (Figure 3B).

Caspase cleavage of tricellulin liberates a fragment of 71 C-terminal amino acids containing a region known to form a coiled-coil dimer [10]. This fragment was not detectable, even not when using high percentage SDS-PAGE gels, either because this small fragment escaped detection on Western blots or due to further degradation. Based on our observations, the antibody used in this study appears to bind to this fragment since truncated tricellulin where the C-terminus is deleted after D487 was no longer detectable. Definitively, it is worth testing by other methods if this fragment is detectable and of functional relevance. Not only due to its size can it easily translocate to the nucleus where it might be involved in transcriptional regulation, as shown for the amyloid precursor protein intracellular domain (AICD) [44] or the Notch intracellular domain (NICD) [45]. It has also been reported that nuclear localization of full-length tricellulin in pancreatic cancer promotes cell proliferation and invasiveness [46].

Based on our studies, we cannot exclude that the remaining tricellulin N-terminal part undergoes further processing steps as observed for occludin. There, the induction of apoptosis leads to caspase cleavage in the cytosolic C-terminus and to further fragmentation by metalloproteinases probably in the first extracellular loop [35]. First evidence for a potential contribution of metalloproteinases is provided by a study in Caco2 cells, where MMP-2- and MMP-3-catalyzed degradation of tricellulin is induced by N-3-(oxododecanoyl)-homoserine lactone (C12-HSL) [47].

A consequence of caspase cleavage and the concomitant release of the tricellulin C-terminal fragment is the disruption of the ZO-protein-mediated linkage of tricellulin to the actin cytoskeleton. Moreover, loss of the C-terminal amino acids might impair localization of tricellulin to tTJs by LSR/angulin-1. However, we observed that also LSR/angulin-1 is fragmented after induction of apoptosis. We conclude that, during apoptosis, tTJs are targeted at more than one level.

Taken together, here we showed that tricellulin is efficiently targeted by caspases in apoptotic cells within its C-terminal coiled-coil domain at D487 and D441. This together with concomitant cleavage of LSR/angulin-1 may contribute to a coordinated release of apoptotic cells from cell layers as it occurs continuously in the gastrointestinal tract at high frequency.

## 4. Materials and Methods

### 4.1. Cell Culture

Madine–Darby canine kidney II (MDCKII) cells were cultured in MEM with 10% (*v/v*) FBS and 1% (*v/v*) penicillin/streptomycin. Human bladder carcinoma cells (RT-112) as well as human embryonal kidney-293 cells (HEK-293) were cultivated in DMEM with 10% (*v/v*) FBS and 1% (*v/v*) penicillin/streptomycin using standard procedures as described elsewhere [31].

### 4.2. Reagents and Antibodies

Staurosporine was obtained from AppliChem GmbH (Darmstadt, Germany), caspase inhibitors Z-VAD-FMK and Z-DEVD-FMK as well as recombinant active caspase-3 were obtained from BD Biosciences (Heidelberg, Germany). Monoclonal anti-FLAG^®^ M2 antibody was purchased from Sigma-Aldrich (Taufkirchen, Germany), anti-tricellulin (clone 54H19L38) ABfinity™ rabbit monoclonal antibody was from ThermoFisher Scientific (Darmstadt, Germany), anti-LSR (D3E3N) and anti-βactin (8H10D10) antibodies were obtained from Cell Signaling Technology (Frankfurt am Main, Germany) and anti-PARP (Ab-2) antibody was from Calbiochem (#AM30) (Merck KGaA, Darmstadt, Germany). Rabbit anti-GST antibody was provided by Jürgen Wienands. Goat anti-mouse-HRP and goat anti-rabbit-HRP antibodies were purchased from Sigma-Aldrich (Taufkirchen, Germany) and Alexa-Fluor™594-labeled secondary antibody was obtained from Molecular Probes (ThermoFisherScientific, Darmstadt, Germany). Dilution of the antibodies is summarized in Table 1.

### 4.3. Plasmids, Site-Directed Mutagenesis and Transient Transfections

Plasmids were cloned by standard procedures and verified by sequencing. The cloning of p3xFLAG-CMV10-Tric and p3xFLAG-CMV10-Tric∆C constructs was described previously [15]. To generate caspase cleavage-site mutated tricellulin variants, site-directed mutagenesis of the potential caspase cleavage-sites at positions D441 and D487 to asparagine was performed by SBOE (splicing by overlap extension)-PCR. Oligonucleotides used for the SBOE-PCR reactions are summarized in Table 2. The mutated tricellulin cDNAs were cloned into the vector p3xFLAG-CMV10 (Sigma-Aldrich, Taufkirchen, Germany) using *BamH*I restriction site. The C-terminal tricellulin deletion constructs Tric∆441–558 and Tric∆487–558 were generated by PCR using the oligonucleotides 5′-CGG ATC CTC AAA TGA TGG AAG ATC CAG-3′ as forward primer and 5′-CGG ATC CTT AGG GCA TCA CGA TAG GTT TAG-3′ or 5′-GGA TCC TTA CAG CTC ATC AAA CTT CCT CA-3′ as reverse primers, respectively, and cloned into the *BamH*I restriction site of p3xFLAG-CMV10 (Sigma-Aldrich, Taufkirchen, Germany). For recombinant expression of the tricellulin C-terminal cytosolic domain, pGEX4T1-TricC described previously [31] was used. The caspase-site mutated variants of GST-TricC were constructed by PCR employing oligonucleotides 5′-GCG GGA TCC ATG TGG AGG CAT GAG GCA GCT C-3′ and 5′-GCG GGT ACC GGA TCC TTA AGA ATA ACC TTG TAC ATC-3′ using p3xFLAG-CMV10-Tric-D441N, -D487N or -D441N/D487N as templates. After digestion with *BamH*I, the amplified products were ligated into *BamH*I-cleaved and dephosphorylated pGEX4T1 (GE Healthcare, Freiburg, Germany). pCAGGS-LSR for expression of GFP-tagged LSR was obtained from Mikio Furuse and described in [17].

MDCKII and HEK-293 cells were transiently transfected with the indicated plasmids using a DNA:PEI (poyethylenimine) ratio of 1:4 as described previously [48]. Cells were seeded in 6-well plates (2 × 10^5^ cells for MDCKII and 5 × 10^5^ HEK-293 cells per well one day before transfection. For transiently transfected HEK-293 or MDCKII cells, one or two 6-wells were lysed per sample. Empty vectors were transfected as a control.

### 4.4. Induction of Apoptosis and Preparation of Cell Lysates

MDCKII cells were plated on 6-well plates (2 × 10^5^ cells per well for transfection; 8 × 10^5^ cells per well without transfection) or 12-well plates (4 × 10^5^ cells per well) and optionally transfected as described above. One day after seeding or transfection, apoptosis was induced by treatment with 1 µM staurosporine. Cells were lysed at the indicated time points. For inhibition of caspase activity, 10 µM Z-VAD-FMK or 20 µM Z-DEVD-FMK were added 1 h before induction of apoptosis for 6 h. Both floating and adherent cells from one well were harvested and pooled. Cells were lysed with 80 µL (12-well) or 100/150 µL (6-well) ice-cold modified RIPA-buffer (50 mM Tris/HCl pH 7.5, 150 mM NaCl, 0.5% (*w/v*) sodium-deoxycholate, 0.1% (*v/v*) SDS, 1% (*w/v*) Nonidet P-40 including Complete™ protease inhibitor mix (Roche Life Science, Mannheim, Germany)) for at least 15 min on ice. After sonication (15 pulses; cycle 0.5; 70% amplitude; UP100H ultrasound processor, Hilscher Ultrasound Technology, Teltow, Germany) the lysates were centrifuged (10 min, 20,800× *g*, 4 °C) to remove insoluble cell components.

### 4.5. In Vitro Caspase Cleavage

Recombinant glutathione S-transferase (GST)-tagged c-cytosolic (amino acids 366–558) domain of tricellulin wild type and mutant (D441N; D487N; D441N/D487N) proteins were expressed and purified as reported elsewhere [49]. In total, 4 µg of recombinant protein was digested with 150 ng of recombinant, active caspase-3 (BD Biosciences, Heidelberg, Germany) in 20 mM Pipes pH 7.2, 100 mM NaCl, 1 mM EDTA, 10% (*w/v*) sucrose, 10 mM DTT and 0,1% (*w/v*) CHAPS at 37 °C for the indicated incubation times. For inhibition of the in vitro caspase cleavage, 10 µM of pan-caspase inhibitor Z-VAD-FMK or DMSO as a control was added. Finally, samples were analyzed by SDS-PAGE and Western blot analysis.

### 4.6. Cell Lysis and Co-Immunoprecipitation

HEK-293 cells were seeded in 6-well plates and transfected (1µg DNA each construct) with PEI as described above. Cells (two 6-wells) were harvested 48 h after transfection and lysed in 300 µL ice-cold modified RIPA-buffer including Complete™ protease inhibitor mix (Roche Life Science, Mannheim, Germany) for 20 min on ice. After lysis, samples were centrifuged (10 min, 20,800× *g*, 4 °C) to remove insoluble material. For co-immunoprecipitation experiments, 200 µL lysate was incubated with 2 µg of anti-FLAG^®^ M2 antibody for 1 h at 4 °C under constant rotation. Subsequently, 30 µL of equilibrated Protein A Sepharose CL-4B beads (GE Healthcare, Freiburg, Germany) were added and incubated for further 30 min. Finally, beads were washed for three times with lysis buffer and protein complexes were eluted using 2 × SDS-loading buffer at 95 °C for 5 min. Samples were separated by SDS-PAGE and subsequently analyzed by Western blotting. For each experiment, 10 µL of cell lysate (5% of input) was loaded as a control.

### 4.7. Western Blot Analysis

Cell lysates were separated by SDS-PAGE and transferred to polyvinylidene difluoride (PVDF) membrane by tank blotting. After blocking with 5% (*w/v*) dry milk in Tris-buffered saline/Tween20 (TBS-T) for 1 h, membranes were incubated with the primary antibodies overnight at 4 °C and with horseradish peroxidase (HRP)-labeled secondary antibody at least for 1 h at room temperature (Table 1).

### 4.8. Immunofluorescence Microscopy

For immunofluorescence microscopy, MDCKII cells were seeded on transwell filter supports (Millicell Cell Culture Insert, 12 mm, 0.4 µm, Merck Millipore, Darmstadt, Germany) with a density of 150,000 cells/cm^2^. At day 7, cells were treated with 1 µM staurosporine or DMSO for 4 h and 7 h. Subsequently, the cells were fixed with 2% paraformaledhyde in PBS and stained with 1 μg/mL anti-tricellulin (clone 54H19L38) antibody for 18 h at 4 °C followed by secondary antibody incubation (donkey anti-rabbit, Alexa-594 conjugated) for 1 h at room temperature. Nuclei were labeled using DAPI (Sigma-Aldrich, Taufkirchen, Germany). Images (z-stacks) were acquired with a laser scanning system TC/SP5 (Leica Microsystems, Wetzlar, Germany) and were maximum intensity projected.

## Figures and Tables

**Table 1:** Antibodies used in the presented study including dilutions for Western blotting, immunofluorescence microscopy and immunoprecipitation.

Antibody	Dilution	Target	Species
anti-FLAG^®^ M2	WB (1:5.000); IP 2 μg	FLAG-tag	mouse mab
anti-GST	WB (1:20.000)	glutathione-S-transferase	rabbit pab
anti-LSR (D3E3N) XP^®^	WB (1:1.000)	LSR	rabbit mab
anti-PARP-1 (Ab-2)	WB (1:1.000)	PARP	mouse mab
anti-tricellulin (54H19L38) ABfinity™	WB (1:1.000) IF (1 μg/mL)	tricellulin	rabbit mab
anti-βactin	WB (1:1000)	βactin	mouse
goat anti-mouse-HRP	WB (1:50.000–1:100.000)	mouse IgG	goat
goat anti-rabbit-HRP	WB (1:50.000–1:100.000)	rabbit IgG	goat
donkey anti-rabbit-Alexa594	IF (1: 1.000)	rabbit IgG	donkey

**Table 2:** Sequences of oligonucleotides used to generate mutated tricellulin constructs. The bases marked in red represent those bases that differ from the wild-type sequence to generate the indicated mutations.

Mutation	Product	Sequence Oligonucleotides
	1	5′-GCG GGT ACC GGA TCC GCC GCC ATG TCA AAT GAT GGA AGA TCC-3′ 5′-CAC ATA GTT GGG CAT CAC GAT-3′
Tric-D441N	2	5′-ATC GTG ATG CCC AAC TAT GTG-3′ 5′-GCG GGT ACC GGA TCC TTA AGA ATA ACC TTG TAC ATC-3′
	1 + 2 (SBOE)	5′-GCG GGT ACC GGA TCC GCC GCC ATG TCA AAT GAT GGA AGA TCC-3′ 5′-GCG GGT ACC GGA TCC TTA AGA ATA ACC TTG TAC ATC-3′
	1	5′-GCG GGT ACC GGA TCC GCC GCC ATG TCA AAT GAT GGA AGA TCC-3′ 5′-CAC TGC ATT CAG CTC ATC AAA-3′
Tric-D487N	2	5′-TTT GAT GAG CTG AAT GCA GTG-3′ 5′-GCG GGT ACC GGA TCC TTA AGA ATA ACC TTG TAC ATC-3′
	1 + 2 (SBOE)	5′-GCG GGT ACC GGA TCC GCC GCC ATG TCA AAT GAT GGA AGA TCC-3′ 5′-GCG GGT ACC GGA TCC TTA AGA ATA ACC TTG TAC ATC-3′

## Supplementary Materials

Supplementary materials can be found at https://www.mdpi.com/1422-0067/20/19/4882/s1.

## Abbreviations

bTJ	Bicellular tight junction
CHAPS	3-[(3-cholamidopropyl)dimethylammonio]-1-propanesulfonate
DAPI	4,6-diamidino-2-phenylindole
DMEM	Dulbecco’s Modified Eagle’s Medium
DMSO	Dimethyl sulfoxide
EDTA	Ethylenediaminetetraacetic acid
FBS	Fetal bovine serum
GFP	Green-fluorescent protein
GST	Glutathione S-transferase
HRP	Horseradish peroxidase
LSR	Lipolysis-stimulated lipoprotein receptor
mab	Monoclonal antibody
MDCK	Madin-Darby canine kidney cells
pab	Polyclonal antibody
PARP	Poly [ADP-ribose] polymerase
PEI	Polyethlenimine
RIPA	Radioimmunoprecipitation Assay
SBOE-PCR	Splicing by overlap extension PCR
SDS	Sodium dodecyl sulfate
SDS-PAGE	Sodium dodecyl sulfate polyacrylamide gel electrophoresis
TBS-T	Tris-buffered saline/Tween 20
TER	Transepithelial resistance
TJ	Tight junction
tTJ	Tricellular tight junction
ZO-1	Zona occludens-1
Z-DEVD-FMK	N-Benzyloxycarbonyl-Asp-Glu-Val-Asp(OMe) fluoromethylketone
Z-VAD-FMK	N-Benzyloxycarbonyl-Val-Ala-Asp(OMe) fluoromethylketone
