# Supplementary material for: Apoptotic Fragmentation of Tricellulin
Source: Int J Mol Sci. 2019 Oct 1;20(19):4882. doi: 10.3390/ijms20194882 (PMC6801678; doi:10.3390/ijms20194882)
Supplement: Supplementary file 1 [file ijms-20-04882-s001.pdf]

Supplementary Figures

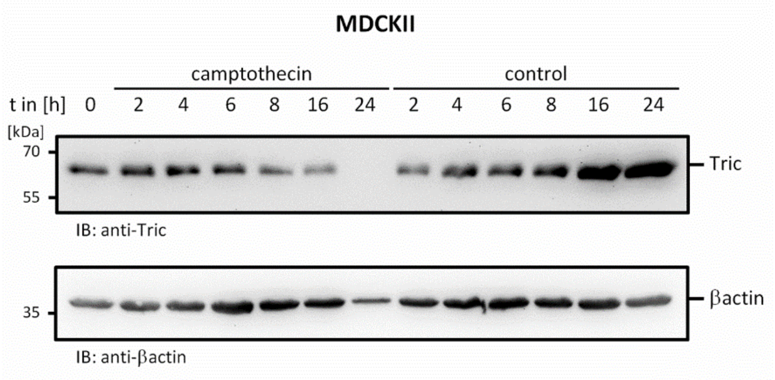

Figure S1. Cleavage of tricellulin after induction of apoptosis with campththecin. MDCKII cells were treated with 5 μM camptothecin or DMSO as a solvent control for the indicated times. Cell lysates were analyzed by Western blotting using anti-tricellulin and anti-βactin antibodies.

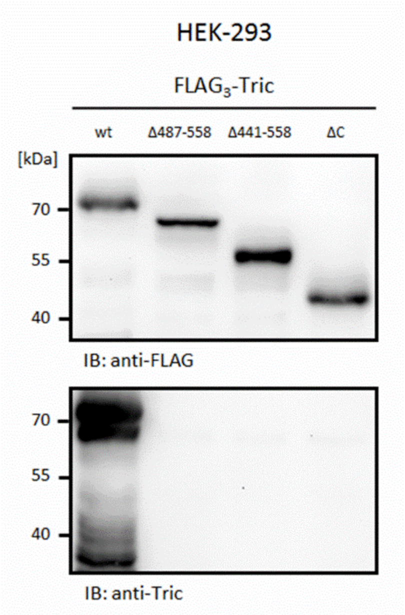

Figure S2. Characterization of anti-tricellulin (clone 54H19L38) ABfinity™ rabbit monoclonal antibody. Tricellulin with deleted amino acids 487–558 is no longer detectable by the anti-tricellulin antibody.
